# Urban forest plant diversity affects soil organic carbon by regulating functional genes in Nanning

**DOI:** 10.1016/j.isci.2026.114856

**Published:** 2026-01-29

**Authors:** Wei Zhou, Zhao Wei, Ning Feng, Mi Luo, Bingpeng Qu, Qiren Luo, Jianbing Zhang

**Affiliations:** 1 Key Laboratory of Environment Change and Resources Use in Beibu Gulf, Ministry of Education, Nanning Normal University, Nanning 530001, China; 2 Guangxi Key Laboratory of Earth Surface Processes and Intelligent Simulation, Nanning Normal University, Nanning 530001, China; 3 School of Environmental and Life Sciences, Nanning Normal University, Nanning 530001, China; 4 School of Geography and Planning, Nanning Normal University, Nanning 530001, China; 5 Guangxi Zhuang Autonomous Region State-owned Qipo Forest Farm, Nanning 530001, China

**Keywords:** natural sciences, environmental science, ecology, plant ecology, soil ecology

## Abstract

Urban forests serve as reservoirs of soil organic carbon (SOC) within ecosystems and play a critical role in the carbon (C) cycle. However, the regulatory mechanisms underlying SOC dynamics across urbanization gradients in urban forests remain poorly understood. In this study, we investigated the interrelationships among plant diversity, SOC and its oxidation-stable fractions, and functional genes across an urbanization gradient encompassing an urban center (UC), subcenter, suburb, and exurb. Our findings revealed that urbanization significantly enhanced plant diversity and altered SOC dynamics. UC exhibited higher plant diversity and increased concentrations of active SOC fractions. Although urbanization did not significantly affect microbial α-diversity, it selectively enriched the relative abundance of specific taxa. C sequestration-related functional genes, particularly K15635 and K01965, were strongly associated with the regulation of SOC dynamics in the urban forest. Our findings underscore the importance of conserving diverse tree communities to enhance the C-sequestration function of urban forests.

## Highlights

• Plant diversity was higher in urban centers than in exurban areas
• Urbanization did not significantly affect microbial diversity or community composition
• Microbial functional genes regulate soil organic carbon sequestration in urban forests
• Urban plant diversity influences active, but not stable, soil organic carbon fractions

## Graphical abstract

**Figure:**
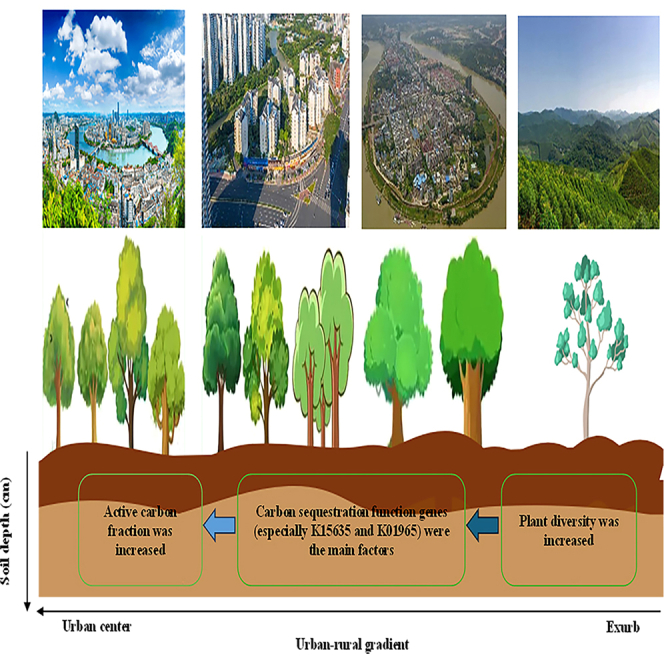

## Introduction

Urbanization has emerged as a critical global issue.^1^^,^^2^ Urban sprawl is projected to continue altering land-use patterns and position cities as major sources of CO_2_ emissions. This process has progressively transformed natural ecosystems into urbanized landscapes, thereby threatening the ecosystem services provided by urban biodiversity.^3^^,^^4^ Urban forests offer essential opportunities for human-nature interaction and are vital for public health. Plant diversity plays a pivotal role in sustaining the functionality and stability of these ecosystems.^5^^,^^6^^,^^7^

Rapid urbanization alters plant community composition through anthropogenic disturbances, thereby disrupting plant-soil feedback mechanisms that maintain ecosystem stability. Although high plant diversity is known to enhance carbon (C) storage in natural ecosystems,^3^^,^^8^^,^^9^ it remains uncertain whether tree diversity in urban environments significantly influences soil organic carbon (SOC) storage. Urbanization gradients, management practices, and other factors create complex plant-soil feedback mechanisms that govern SOC patterns.^4^^,^^10^^,^^11^ Consequently, a clear understanding of the mechanisms linking urban plant diversity and SOC sequestration remains elusive.

Microorganisms are the primary drivers of biogeochemical processes that mediate SOC and nutrient cycling and are indispensable for maintaining the health and functional integrity of urban ecosystems.^7^^,^^12^^,^^13^ Urbanization can reshape soil microbial communities; however, the influence of plant diversity on the stability and functional resilience of these communities in urban ecosystems remains unclear.^6^^,^^7^ Emerging evidence suggests that microbial functional genes, rather than community diversity alone, may be the primary drivers of SOC sequestration in soil.^14^ Previous studies have primarily focused on microbial diversity and community composition within plant-soil-microbe feedback systems; however, a comprehensive understanding of microbial functional genes remains lacking.^15^^,^^16^^,^^17^ Therefore, a key knowledge gap exists in linking urban plant diversity to SOC dynamics via the regulation of microbial functional gene expression.

Nanning, a rapidly urbanizing subtropical city in southern China, provides an ideal setting for examining plant-soil-microbe interactions along an urbanization gradient. As urban expansion intensifies in eastern Nanning, understanding how plant diversity regulates SOC sequestration through microbial mechanisms is particularly relevant to subtropical urban forests. In this study, we examined the influence of plant diversity on SOC storage and its microbial regulatory mechanisms along an urban-rural gradient in eastern Nanning, China. Specifically, our study addressed two questions: (1) How does plant diversity affect SOC storage through direct and indirect pathways mediated by soil microbes? (2) What factors govern plant-soil symbiosis in urban forests? We hypothesized that increased plant diversity would enhance SOC sequestration not by altering the overall microbial diversity but by upregulating specific microbial functional genes involved in C processing. The outcomes of this study are expected to enhance our understanding of urban ecosystem functioning, support the conservation of tree diversity, and guide future strategies for sustainable urban forest planning and management.

## Results

### Tree species distribution, diversity, and structure

In total, 473 individual trees representing 49 species, 39 genera, and 22 families were recorded (Table S1). *Eucalyptus robusta* was the dominant species, comprising 25.79% (*n* = 122) of all individuals, and occurred predominantly at exurban zone (EX) sites. The next most abundant taxa were *Bauhinia purpurea* (*n* = 37), *Cassia siamea* (*n* = 33), *Ficus macrocarpa* (*n* = 25), *Taxodium distichum* (*n* = 22), and *Eucalyptus saligna* (*n* = 20).

The mean tree height ranged from 3 to 12 m, with *Dracontomelon duperreanum* reaching a maximum height of 12 m. *Osmanthus fragrans* var. *thunbergii*, *Delonix regia*, *Cerasus campanulata*, and *Ficus cyathistipula* exhibited a minimum height of 3 m. The mean diameter at breast height (DBH) and crown area ranged from 9.55 to 101.85 cm and 1 to 200 m^2^, respectively. *Ficus elastica* exhibited the greatest mean DBH (101.86 cm) and crown area (200 m^2^), whereas *Trachycarpus fortunei* had the smallest mean DBH (9.55 cm) and *Caryota mitis* and *Alstonia scholaris* had the smallest crown areas (1 m^2^ each).

**Figure 1:**
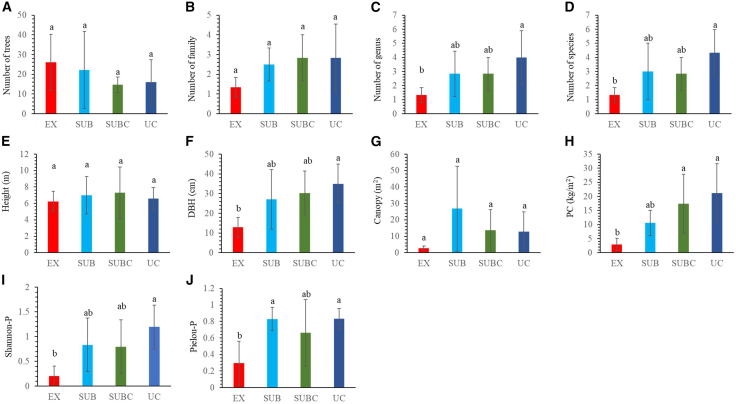
Differences in tree diversity and structure (A) Number of trees. (B) Number of families. (C) Number of genera. (D) Number of species. (E) Tree height. (F) DBH. (G) Crown breadth (canopy). (H) Plant C stock (PC). (I) Plant Shannon-Wiener index (Shannon-P). (J) Plant Pielou index (Pielou-P). Different letters indicate significant (*p* < 0.05) differences among the treatments. Error bars represent standard deviation (*n* = 6).

Along the urbanization gradient, no significant differences were observed in individual density (15–26 trees per plot), family richness (1.33–2.83), mean height (6.24–7.29 m), or crown area (2.87–26.81 m^2^). In contrast, genus richness (1.33–4), species richness (1.33–4.33), mean DBH (12.96–34.94 cm), and plant C storage (2.98–21.24 kg m^−2^) were significantly greater in the urban center (UC) than in the EX (*p* < 0.05). Similarly, plant diversity indices, including species richness and H′, were markedly higher in UC (4.33, 1.19) than in EX (1.33, 0.21) (*p* < 0.05). Overall, the plant C stock exhibited a clear increasing trend from the exurb to the UC (Figure 1).

### SOC and its fractions

**Figure 2:**
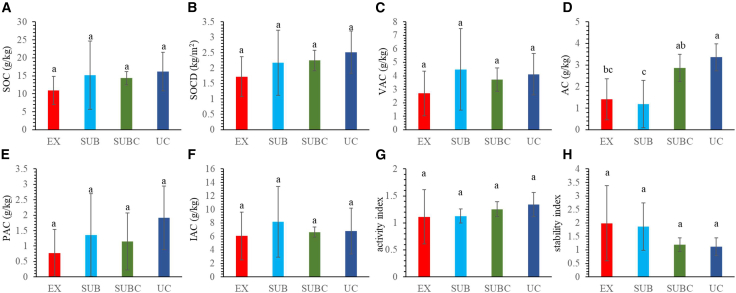
Differences in SOC, C fractions, and C indices (A) SOC. (B) SOCD. (C) VAC. (D) AC. (E) PAC. (F) IAC. (G) AI. (H) SI. Different letters indicate significant (*p* < 0.05) differences among treatments. Error bars represent standard deviation (*n* = 6).

Except for the active SOC (AC) fraction, the SOC (10.95–16.19 g/kg), SOC density (SOCD; 1.72–2.51 kg/m^2^), very labile SOC (VAC) (2.70–4.47 g/kg), partially active SOC (PAC) (0.77–1.91 g/kg), and inactive SOC (IAC) (6.06–8.17 g/kg) fractions did not differ significantly along the urbanization gradient (*p* > 0.05; Figure 2).

The AC content in the UC (3.36 g/kg) was significantly higher than that in the EX (1.41 g/kg) and suburban zone (SUB) (1.19 g/kg) zones (*p* < 0.05). The activity index (AI) showed an increasing trend (1.11–1.34), whereas the stability index (SI) decreased (1.98–1.12) along the urbanization gradient; however, neither change was statistically significant (*p* > 0.05).

### Microbial diversity and community structure

**Figure 3:**
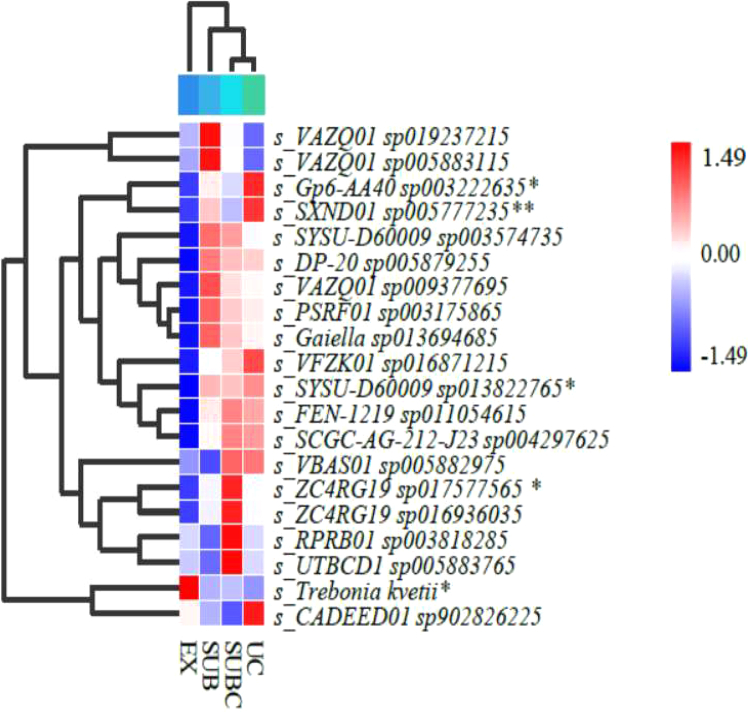
Heatmap of differential soil microbial communities (species level) Statistical significance was determined by one-way ANOVA followed by Tukey honestly significant difference (HSD) post hoc test. “∗” and “∗∗” indicate significant (*p* < 0.05) and very significant (*p* < 0.01) differences among treatments, respectively.

Figure 3 illustrates the clustering of the top 20% most abundant microbial taxa, selected at the species level, to focus on the dominant community members most likely to influence bulk ecosystem processes and reduce noise from rare taxa along the urbanization gradient. Compared with the EX, the UC showed significantly elevated abundances of Gp6-AA40 (*p* < 0.05), SXND01 (*p* < 0.01), SYSU-D60009 (*p* < 0.05), and ZC4RG19 (*p* < 0.05), whereas the abundance of *Trebonia kvetii* was significantly reduced (*p* < 0.05).

**Figure 4:**
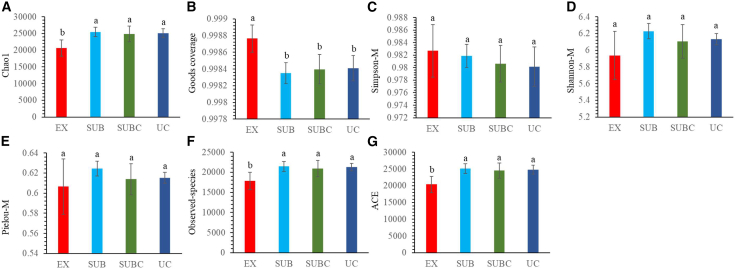
Differences in microbial diversity across EX, SUB, urban subcenter (SUBC), and TDC (A) Microbial Chao1 index. (B) Microbial Goods coverage index. (C) Microbial Simpson index (Simpson-M). (D) Microbial Shannon-Wiener index (Shannon-M). (E) Microbial Pielou index (Pielou-M). (F) Microbial observed-species index. (G) Microbial ACE index. Different letters indicate significant differences among treatments (*p* < 0.05). Error bars represent the standard deviation (*n* = 6).

Chao1, observed species richness, and ACE were significantly lower in the EX than in UC (*p* < 0.05), whereas Good’s coverage was significantly higher in the EX (*p* < 0.05). The Simpson, Shannon, and Pielou indices did not differ significantly between UC and EX. Taken together, these α-diversity metrics (Chao1, observed species richness, and ACE) indicate that microbial richness, but not necessarily overall diversity (Simpson, Shannon, and Pielou), tends to increase with urbanization (Figure 4).

**Figure 5:**
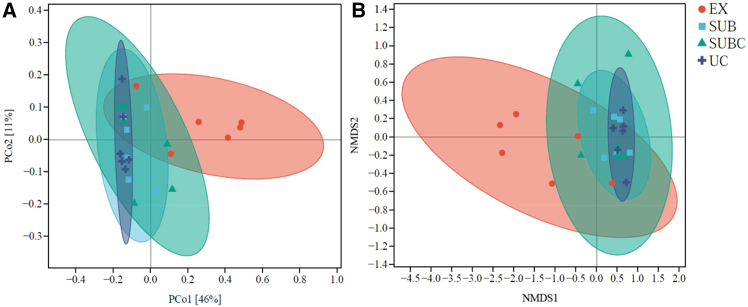
PCoA and NMDS analyses of soil microbial communities across EX, SUB, SUBC, and UC (A) PCoA. (B) NMDS.

Principal coordinate analysis (PCoA) and non-metric multidimensional scaling (NMDS) both revealed a pronounced separation in β-diversity between UC and EX soils (Figure 5). The first two PCoA axes explained 46% and 11% of the total variance, respectively. Consistent with these patterns, ANOSIM based on Bray-Curtis dissimilarity confirmed significant differentiation between the zones (R^2^ = 0.35, *p* < 0.01).

### Microbial functional genes

**Figure 6:**
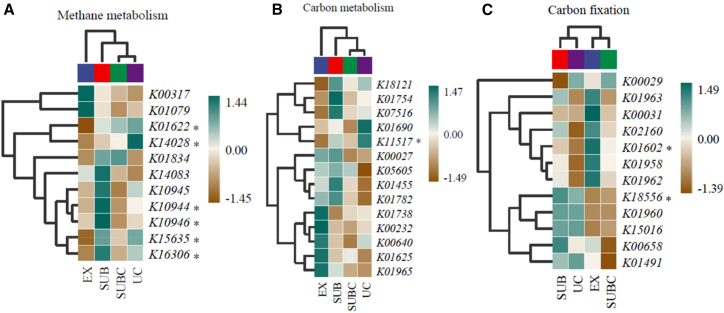
Differential analysis of microbial functional genes Heatmaps showing the relative abundance of KEGG orthologs involved in (A) methane metabolism genes and (B) C metabolism genes. (C) C fixation genes. Statistical significance was determined by one-way ANOVA followed by Tukey HSD post hoc test. “∗” indicates significant differences among treatments (*p* < 0.05).

Functional genes associated with methane metabolism, C fixation, and C metabolism were successfully detected in all samples (Figure 6). Compared with the EX, the UC exhibited significantly higher abundance of the functional genes K01622, K14028, K15635, and K11517 (*p* < 0.05). Conversely, the abundance of K01602 was significantly lower in UC (*p* < 0.05).

### Relationship between plant SOC and soil microbial diversity, taxa, and functional genes

**Figure 7:**
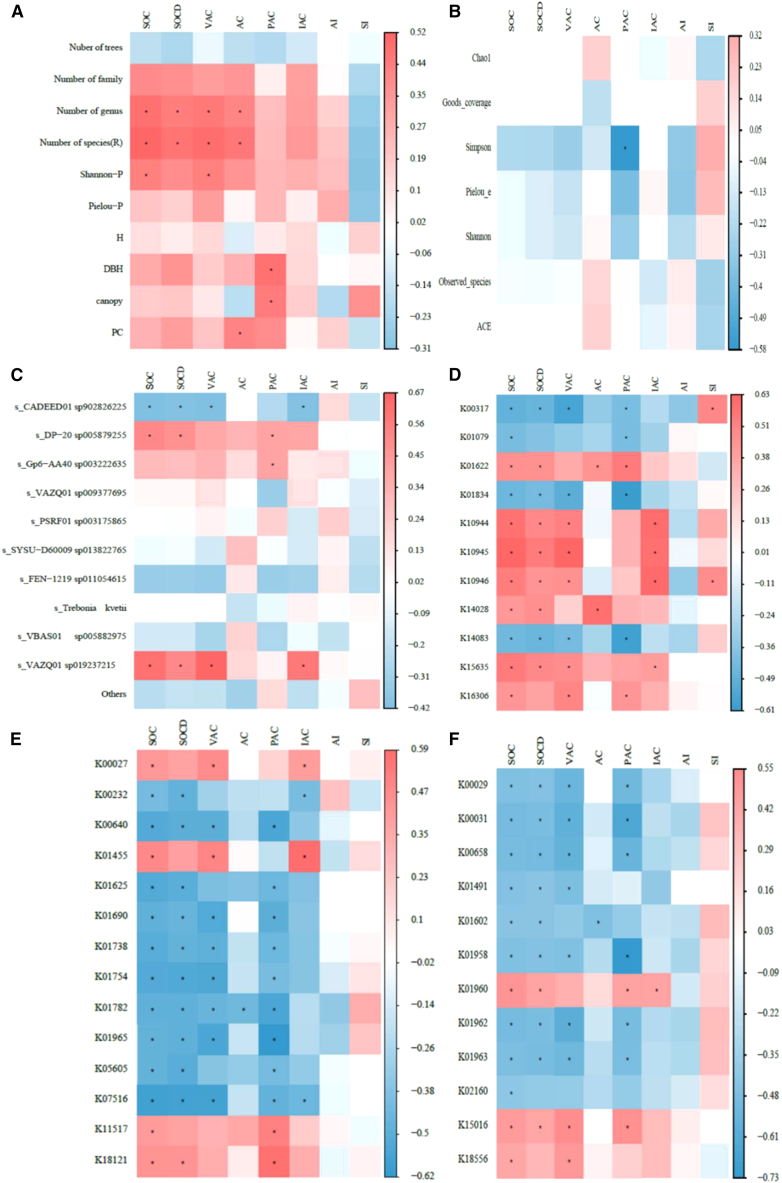
Pearson correlation analysis Pearson correlation between (A) plant factors, (B) microbial diversity, (C) microbial community, (D) methane metabolism genes, (E) C metabolism genes, (F) C fixation genes, and SOC factors. “∗” indicates a significant correlation at the 0.05 level.

The Pearson correlation coefficients are shown in Figure 7. Genus and species richness exhibited significant positive correlations with SOC, SOCD, and active C fractions (VAC and AC) (*p* < 0.05). H′ was also positively correlated with SOC and VAC (*p* < 0.05). In addition, the mean DBH and crown area were significantly and positively correlated with PAC (*p* < 0.05).

Microbial α-diversity indices were generally not correlated with SOC and its fractions, except for a negative correlation between the Simpson index and PAC (*p* < 0.05). At the taxonomic level, *CADEED01* sp. 902826225 exhibited significant negative correlations with SOC, SOCD, VAC, and IAC (*p* < 0.05), whereas *DP-20* sp. 005879255 and *VAZQ01* sp. 019237215 exhibited significant positive correlations with SOC-related variables (*p* < 0.05).

Most C sequestration genes were significantly associated with SOC and its fractions (*p* < 0.05). In contrast, plant diversity indices were not significantly correlated with overall microbial diversity or community composition (*p* > 0.05), except for *DP-20*sp. 005879255. Notably, genes K01965 and K15635 were associated with both SOC and plant diversity (Figure S2; Table S2–S4).

**Figure 8:**
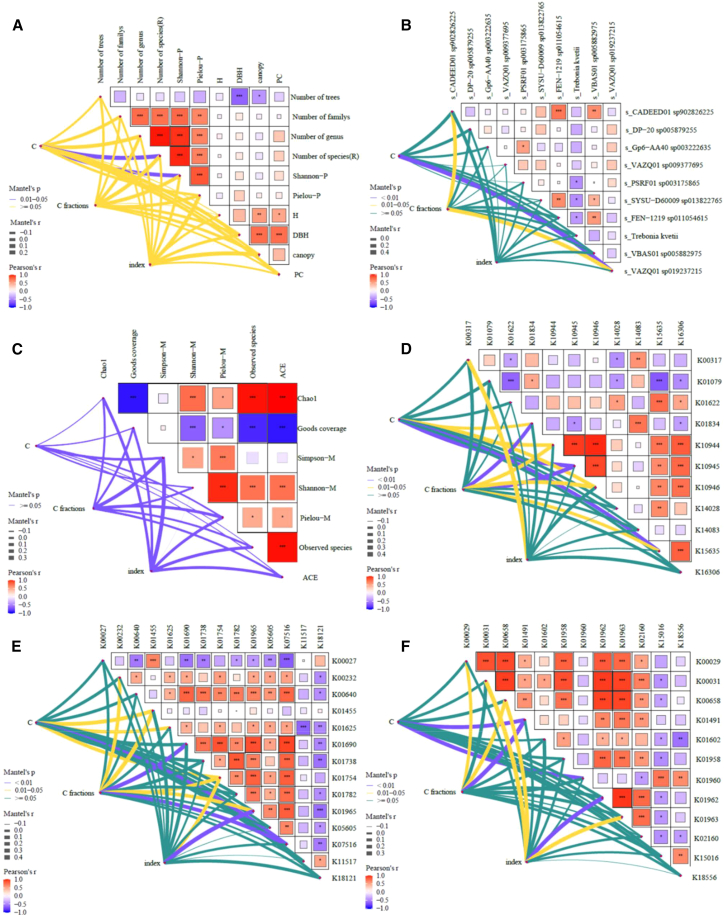
Mantel test Mantel test between (A) plant factors, (B) microbial diversity, (C) microbial community, (D) methane metabolism genes, (E) C metabolism genes, (F) C fixation genes, and SOC factors. The color of the line indicates the significance level, and the width of the line is proportional to the Mantel R value. “∗” , “∗∗”, and “∗∗∗”indicates a significant correlation at the 0.05, 0.01, and 0.001 level, respectively.

Species richness (R = 0.26) and the plant Shannon index (R = 0.19) were positively correlated with SOC (*p* < 0.05). VAZQ01 sp019237215 (R = 0.55) was significantly and positively correlated with SOC (*p* < 0.01), and CADEED01 sp902826225 (R = 0.38) was positively correlated with C fractions (*p* < 0.05). In methane metabolism, K01834, K10944, K10945, K10946, and K15635 were significantly or highly significantly positively correlated with SOC and C fractions. Regarding C fixation, K01491 (R = 0.34 and 0.40, respectively) was highly significantly positively correlated with SOC and C fractions (*p* < 0.01); K01960 (R = 0.31) was also highly significantly positively correlated with SOC (*p* < 0.01); K00029 (R = 0.18) and K00658 (R = 0.28) were significantly positively correlated with C fractions (*p* < 0.05). In addition, K00031 (R = 0.40), K00658 (R = 0.29), K01962 (R = 0.37), and K01963 (R = 0.32) were significantly or highly significantly positively correlated with the C index (*p* < 0.05 or *p* < 0.01). In C metabolism, K01754 (R = 0.23, 0.26, and 0.24, respectively) was significantly positively correlated with SOC, C fractions, and the C index (*p* < 0.05), and K01455 (R = 0.34 and 0.39) and K07516 (R = 0.32 and 0.30) were significantly or highly significantly positively correlated with SOC and C fractions. K01965 (R = 0.25 and 0.42) was significantly or highly significantly positively correlated with C fractions. K01625 (R = 0.22) was significantly positively correlated with SOC, and K00232 (R = 0.25) and K01690 (R = 0.36) were significantly (*p* < 0.05) or highly significantly (*p* < 0.01) correlated with C fractions. However, the microbial diversity index was not significantly correlated with C (*p* > 0.05) (Figure 8).

## Discussion

### Urban tree diversity enhances SOC content

Our results demonstrate that urbanization increases tree diversity, which is associated with a higher concentration of active C fractions in urban forest soils. In this study, the tree assemblage in the UC exhibited significantly higher taxonomic diversity than that in the EX. In the EX, the stand was dominated by eucalyptus, which led to a lower mean DBH and reduced species diversity. Recent investigations have also reported greater plant diversity in UC than in EX.^18^^,^^19^ Yang et al.^20^ demonstrated that urbanization enhances the evolutionary distinctiveness of woody flora, whereas Moreno et al.^21^ emphasized the critical role of urban parks in sustaining ecosystem services and biodiversity in urban environments. Large trees (DBH >30 cm) were primarily concentrated in urban parks, whereas the mean DBH in EX was only 12.96 cm. Greater plant diversity can enhance the structural and functional stability of urban forests; consequently, municipalities and urban forest managers should prioritize afforestation in EX to enhance diversity.^22^ One effective strategy is to enrich plantations with native tree species to maximize the ecosystem benefits.^23^ Changes in the composition and diversity of urban forests inevitably alter SOC stocks, particularly in the topsoil.^24^^,^^25^

Accumulating evidence indicates that anthropogenic disturbances exert both direct and indirect effects on SOC storage.^8^^,^^26^ Our results indicate that the number of genera and species positively influences SOC concentrations and its active fractions, suggesting that SOC in UC is inherently more labile and susceptible to anthropogenic perturbations. We posit that increases in species richness enhance the input of plant-derived materials (litter and root exudates) to the topsoil, thereby elevating the abundance of the active fraction of C.^24^^,^^27^^,^^28^ Our findings corroborate previous research identifying tree diversity as a key driver of SOC accumulation in forest ecosystems.^8^ Although numerous studies have identified urban trees as potential C sinks, a standardized protocol for estimating tree biomass and C stocks remains elusive.^23^^,^^26^ In this study, we used general allometric equations to quantify tree C stocks and observed significantly higher values in UC than in EX. This discrepancy arises because urban trees attain larger diameters and, therefore, sequester more C. Moreover, we detected a significant positive correlation between tree C stock and the active C fraction, indicating that urban trees predominantly enhance the labile SOC component. Given that multiple studies have emphasized the importance of large-diameter trees in maintaining the C balance of urban forests, our results support conservation management priorities.^3^ In summary, our findings demonstrate that urbanization enhances plant diversity, with the urban core exhibiting the highest taxonomic richness and exerting pronounced effects on soil C cycling.

### Microbial community diversity shows a limited effect on SOC

Urbanization increased microbial richness and altered β-diversity; however, these overall community measures were poorly correlated with SOC, suggesting a decoupling between general community structure and C storage in this system. From EX to UC, the α-diversity indices (Chao1, observed species, and ACE) increased significantly, whereas the β-diversity analyses revealed a pronounced separation between EX and UC. Grierson et al.^6^ reported elevated bacterial α-diversity in intensively managed parks, supporting these findings. Previous studies have indicated that plant assemblages primarily influence the diversity of fungi in urban parks.^17^ Our data confirm and extend this understanding, showing that plant species composition also modulates the overall microbial community structure and that urbanization positively correlates with microbial richness.

Urban cores undergo intensive management practices, including irrigation, fertilization, soil compaction, and frequent mowing, and experience elevated anthropogenic disturbances, all of which collectively influence microbial diversity.^29^^,^^30^ Predicting ecosystem function, microbial community composition, and functional traits provides more informative insights than α-diversity indices.^6^ Our results revealed minimal shifts in the overall community composition across sampling sites; however, several taxa (*Gp6-AA40* [belongs to the order of Vicinamibacterales], *SXND01* [belongs to the order of Entotheonellales], *SYSU-D60009* [belongs to the order of Dongiales], *ZC4RG19* [belongs to the order of Acidimicrobiales], and *Trebonia kvetii*) exhibited significantly higher or lower abundance in UC. This suggests that the soil microbial communities in eastern Nanning are resilient and maintain stability despite the pressures of urbanization.^25^

Plant-microbe interactions synergistically drive long-term SOC sequestration.^16^^,^^31^ Microbial α-diversity indices showed few associations with SOC, except for a significant negative correlation between the Simpson index and PAC (*p* < 0.05). This finding suggests that microbial community evenness does not directly regulate SOC stocks. Thus, the influence of microbial communities on SOC is multifactorial and context-dependent.^25^^,^^31^ Changes in soil physicochemical properties and vegetation characteristics induced by urbanization are key drivers of microbial community assembly along urbanization gradients.^31^^,^^32^

### Microbial functional genes regulate SOC dynamics

The abundance of specific C-cycling functional genes, particularly K15635 and K01965, was strongly associated with SOC and plant diversity, indicating a potential mechanistic link via the microbial metabolic potential.^33^ Our results showed that urban forests with different plant diversities harbor distinct carbon-cycling functional gene profiles. Plant diversity directly and indirectly influences soil microbial communities. In this study, it affected the abundance of C-sequestration genes without altering the overall microbial α-diversity. These findings suggest that urban tree diversity may enhance SOC primarily through the upregulation of C sequestration genes rather than through shifts in microbial diversity.

Therefore, we investigated the nuanced relationships between C-sequestration functional genes and SOC. Functional genes exhibited heterogeneous responses along the urban-rural gradient, suggesting distinct contributions to SOC cycling. Methane metabolism, C fixation, and C metabolism are central pathways in soil C cycling.^26^^,^^34^^,^^35^ KEGG-annotated genes involved in carbohydrate metabolism served as proxies for the microbial carbon cycling potential. Genes encoding K01622 (1,6-bisphosphate aldolase/phosphatase), K14028 (methanol dehydrogenase), and K15635 (2,3-bisphosphoglycerate-independent phosphoglycerate mutase) were significantly more abundant in urban than in EX, indicating that urbanization alters tree community composition, which in turn modulates the activity of key C-processing pathways. These genes are pivotal for CO_2_ fixation and CH_4_ metabolism, influencing the conversion of atmospheric CO_2_ into soil organic compounds and regulating the C cycle and methane emissions.^36^

Urbanization-induced shifts in microbial communities alter the soil C metabolic capacity; consequently, SOC dynamics are strongly dependent on microbial metabolic activity.^31^ Dai et al.^37^ demonstrated that microbial functional diversity is the primary determinant of the abundance of C- and N-cycling genes, whereas Sun et al.^31^ emphasized the crucial role of these metabolic activities in long-term SOC sequestration. Our data corroborate that C metabolic genes are more closely associated with SOC and its fractions than with the overall microbial community structure, underscoring the central importance of functional genes in urban SOC storage.

Plant diversity significantly influences the abundance of C-functional genes, whereas soil properties appear to have comparatively minor effects.^25^ Mishra et al.^15^ further highlight that plant community structure is more influential than microbial community composition in determining functional gene profiles. Among the identified genes, K01965 (propionyl-CoA carboxylase α-subunit) and K15635 emerged as key regulators of SOC dynamics under the distinct environmental conditions of urban forests. These findings suggest that soil C dynamics in urban forests are more strongly associated with microbial functional gene expression than by overall microbial community structure.

In summary, urbanization enhances the taxonomic diversity of urban-core tree communities, which subsequently increases the active soil C pool and modulates microbial C-cycling processes. Specifically, the abundance of functional genes involved in microbial C cycling is strongly associated with SOC sequestration in urban forests.

### Implications for urban forest studies and management

Although plant diversity in urban forests has been extensively studied, the functional roles of soil microorganisms, particularly beneficial taxa, have largely been overlooked. As urban soils are predominantly anthropogenic substrates, soil types cannot be standardized across different sites. Although our study was limited to a small spatial extent in eastern Nanning, it advances our understanding of the complexity of tree species diversity in urban environments.

The relationship between plant diversity and C sequestration is inherently complex and remains an active area of research.^4^ Disentangling these causal pathways is challenging because the C, N, and P cycles function as interconnected feedback loops rather than unidirectional processes.^38^ Future studies should adopt broader spatial scales and incorporate additional environmental variables.^39^ Overall, our findings provide new insights into the mechanisms underlying plant-SOC-microbial interactions and may support more accurate predictive modeling of soil C dynamics in response to changes in urban plant diversity.^24^

In conclusion, plant diversity increased progressively with urbanization intensity, from EX to UC. The active C fractions exhibited an increasing trend, whereas the stable fractions showed a corresponding decrease in trend. Urbanization did not significantly alter microbial α-diversity or overall community composition, but it selectively shifted the relative abundances of specific taxa, such as Gp6-AA40 (belongs to the order of Vicinamibacterales) and SXND01(belongs to the order of Entotheonellales). Plant diversity indices (genus richness, species richness, and H′) were significantly correlated with the labile SOC fractions. We concluded that C sequestration genes, particularly K15635 and K01965, emerged as the primary determinants of SOC storage (particularly labile fractions) in urban forests, revealing a functional genetic pathway through which tree diversity enhances SOC sequestration.

### Limitations of the study

This study has several limitations. First, despite the well-documented spatial heterogeneity of urban soils, our sampling was confined to a relatively small geographic area with a limited number of sites, potentially constraining the representativeness and broader applicability of our findings. Second, the absence of key biogeochemical metrics such as microbial carbon use efficiency precludes a mechanistic understanding of carbon sequestration processes in urban forest soils. To advance this field, future work should adopt multi-scale, spatially representative sampling strategies across diverse urban gradients, integrating microbial physiology with ecosystem-level carbon dynamics to unravel the drivers and resilience of urban soil carbon sinks.

## Resource availability

### Lead contact

Requests for further information and resources should be directed to and will be fulfilled by the lead contact, Jianbing Zhang (20220601023@nnnu.edu.cn).

### Materials availability

This study did not generate new unique reagents.

### Data and code availability

• Microbial sequencing data have been deposited at the National Center for Biotechnology Information (NCBI) Sequence Read Archives (SRA) under the BioProject number PRJNA1401303 and are publicly available.
• The code in this study is deposited in the online repository at https://github.com/bsstzw/urban-soil-anova-code.
• Any additional information required to reanalyze the data reported in this paper is available from the lead contact upon request.

## Acknowledgments

This work was supported by the Guangxi Natural Science Foundation (2025GXNSFBA069400), Project for Enhancing Young and Middle-aged Teacher's Research Basis Ability in Colleges of Guangxi (2025KY0448), Qingmiao Program of Guanxi (60203038919630214), 10.13039/501100001809National Natural Science Foundation of China (42167040), The Opening Foundation of 10.13039/100020750Guangxi Key Laboratory of Earth Surface Processes and Intelligent Simulation, Nanning Normal University (NNNU-KLOP-K2302), and Innovation and Entrepreneurship Training Program for College Students (202510603051, S202510603179). We would like to thank KetengEdit (www.ketengedit.com) for its linguistic assistance during the preparation of this manuscript.

## Author contributions

Conceptualization, W.Z. and J.Z.; methodology, W.Z. and J.Z.; investigation, Z.W., N.F., M.L., B.Q., and Q.L.; writing—original draft, W.Z.; writing—review & editing, W.Z. and J.Z.; funding acquisition, W.Z., Z.W., N.F., and J.Z.; resources, W.Z. and J.Z.; supervision, J.Z.

## Declaration of interests

The authors declare no competing interests.

## Declaration of generative AI and AI-assisted technologies in the writing process

During the preparation of this work, we used AI (Qwen and Kimi) to polish the language. After using this tool, we reviewed and edited the content as needed and take full responsibility for the content of the publication.

## STAR★Methods

### Key resources table

**Table:** 

REAGENT or RESOURCE	SOURCE	IDENTIFIER
**Deposited data**		

Urban forest soil metagenomic data	This paper	BioProject number PRJNA1401303
Statistical code	This paper	https://github.com/bsstzw/urban-soil-anova-code.

**Software and algorithms**		

R	https://cran.r-project.org/	version 4.5.2
GeneCloud online platform	Shanghai Personal Biotechnology Co.,Ltd.	https://www.genescloud.cn

### Experimental model and study participant details

This study does not include experimental model or study participant.

### Method details

#### Study area and sample collection

Nanning, the capital of the Guangxi Zhuang Autonomous Region (22°12′–24°02′ N, 107°19′–109°38′ E), is characterized by a subtropical monsoon climate with an average annual precipitation of 1,300 mm and an average annual temperature of 21.7°C. Eastern Nanning is undergoing rapid urbanization; accordingly, we selected 18 urban parks and 6 exurban plantations across the eastern region. At each site, a 20 m × 20 m plot was randomly established, and surface soils (0–20 cm) were sampled in July 2024 using a five-point composite sampling design. Although within-site replication was limited to a single plot, the study was designed as a landscape-scale urbanization gradient analysis with replication across the gradient (*n* = 6 sites per urbanization level). The composite soil sample, derived from five points within each plot, integrated small-scale spatial heterogeneity and provided a statistically robust basis for detecting systematic differences among the urbanization levels. All samples were immediately transported to the laboratory and stored at −80 °C pending further analysis. Sampling focused on the topsoil layer (0–20 cm), which is most directly influenced by plant litter inputs, root exudates, and surface management practices, and where microbial activity and labile C dynamics are pronounced.^40^^,^^41^

Ring-road development serves as a reliable proxy for urbanization intensity and has been widely used to assess the impact of urbanization on soil properties.^5^^,^^42^ In this study, we used the ring-road system to delineate four levels of urbanization: areas within the first ring road were designated as the urban center (UC); those between the first ring road and the Na’an Expressway were defined as the urban subcenter (SUBC); those between the Na’an Expressway and the Belt Freeway were classified as the suburban zone (SUB); and areas beyond the Belt Freeway were designated as the exurban zone (EX) (Figure S1).

All trees measuring ≥ 3 m in height within each plot were identified at the family, genus, and species levels. Their structural attributes, including height, diameter at breast height (DBH), and crown area, were recorded in accordance with the vegetation classification protocols outlined in Flora of China (1959–2004).

The Shannon-Wiener index (H′), Pielou's evenness index (J), and plant C stock (PC) were calculated using Equations 1, 2, 3, 4, and 5.^4^^,^^43^^,^^44^

(Equation 1) $$H^{’}=-\sum_{i=1}^{s}P_{i}\;\ln\;P_{i}$$

(Equation 2) $${J=H}^{’}/lnS$$

(Equation 3) $$Above-ground\;biomass\;\left(AGB\right)=34.4703-8.067\;\left(DBH\right)+0.6589\;\left({DBH}^{2}\right)$$

(Equation 4) Below−groundbiomass(BGB)=AGB×0.2

(Equation 5) PC=AGB+BGB

where S is the total number of species, *P*_*i*_ represents the number of trees for species *i*/the total number of trees, and DBH is the diameter at breast height (cm).

#### SOC and its fractions determination

The total SOC concentration was determined using the K_2_Cr_2_O_7_–H_2_SO_4_ wet oxidation method. SOC fractions were quantified using a modified Walkley–Black fractionation method.^45^^,^^46^ Concentrated H_2_SO_4_ volumes of 5, 10, and 20 mL were used to prepare acid solutions with concentrations of 12, 18, and 24 N, respectively. Then, 10 mL of 1 N K_2_Cr_2_O_7_ was added to 1 g of soil, followed by 10 mL of concentrated H_2_SO_4_ for oxidation. Residual K_2_Cr_2_O_7_ was back-titrated using a standardized FeSO_4_ solution.

Four SOC fractions were operationally defined as follows: very labile SOC (VAC), representing C oxidizable by 12 N H_2_SO_4_; AC, defined as the difference in C oxidizable between 18 N and 12 N H_2_SO_4_; partially active SOC (PAC), defined as the difference in C oxidizable between 24 N and 18 N H_2_SO_4_; and inactive SOC (IAC), defined as the difference between total SOC and C oxidizable by 24 N H_2_SO_4_.

These fractions were subsequently grouped into labile (VAC + AC) and recalcitrant (PAC + IAC) categories. The activity index (AI) and stability index (SI) were calculated using Equations 6 and 7, respectively.^1^

(Equation 6) $$AI=\frac{VAC}{SOC}\times 3+\frac{AC}{SOC}\times 2+\frac{PAC}{SOC}\times 1$$

(Equation 7) $$SI=\frac{IAC+PAC}{VAC+AC}$$

#### DNA extraction, metagenome sequencing, and Kyoto Encyclopedia of genes and genomes (KEGG) annotation

Microbial genomic DNA was extracted from 0.5 g of fresh soil using the Mag-Bind Soil DNA Kit (Omega Bio-Tek, M5635-02) according to the manufacturer’s instructions and stored at −20°C until further analysis. DNA quantity and integrity were assessed using a Qubit™ 4 Fluorometer (Invitrogen, USA) and 1% agarose gel electrophoresis, respectively.

Paired-end (PE150) metagenomic libraries with approximately 400 bp inserts were prepared using the Illumina TruSeq Nano DNA LT Library Preparation Kit. The libraries were sequenced on an Illumina NovaSeq- Xplus platform (2 × 150 bp) at Personal Biotechnology Co., Ltd., Shanghai, China. Raw sequencing data were deposited in the National Center for Biotechnology Information (NCBI) Sequence Read Archives (SRA) under the BioProject number PRJNA1401303.

Functional genes were annotated using the KEGG database (http://www.genome.jp/kegg/) to identify the metabolic pathways. Functional annotation and taxonomic classification were performed for all samples to characterize the microbial metabolic potential.

### Quantification and Statistical analysis

Descriptive statistics (mean ± standard deviation) were calculated using Microsoft Excel 2019. Differences among urbanization levels were analyzed using one-way analysis of variance, followed by Tukey HSD post hoc test using R software (version 4.5.2) for plant diversity indices, SOC fractions, microbial α-diversity, and functional gene abundance. “∗” and “∗∗” indicate significant (*P* < 0.05) and very significant (*P* < 0.01) differences among treatments, respectively. The microbial community structure and functional gene profiles were visualized using the GeneCloud online platform (https://www.genescloud.cn). Pearson’s correlation and Mantel tests were performed within the platform.
