# Supplementary material for: Urban forest plant diversity affects soil organic carbon by regulating functional genes in Nanning
Source: iScience. 2026 Jan 29;29(3):114856. doi: 10.1016/j.isci.2026.114856 (PMC12925049; doi:10.1016/j.isci.2026.114856)
Supplement: Document S1. Figures S1 and S2 and Tables S1–S6 [file mmc1.pdf]

iScience, Volume *29*

Supplemental information

Urban forest plant diversity affects soil organic carbon by regulating functional genes in Nanning

Wei Zhou, Zhao Wei, Ning Feng, Mi Luo, Bingpeng Qu, Qiren Luo, and Jianbing Zhang

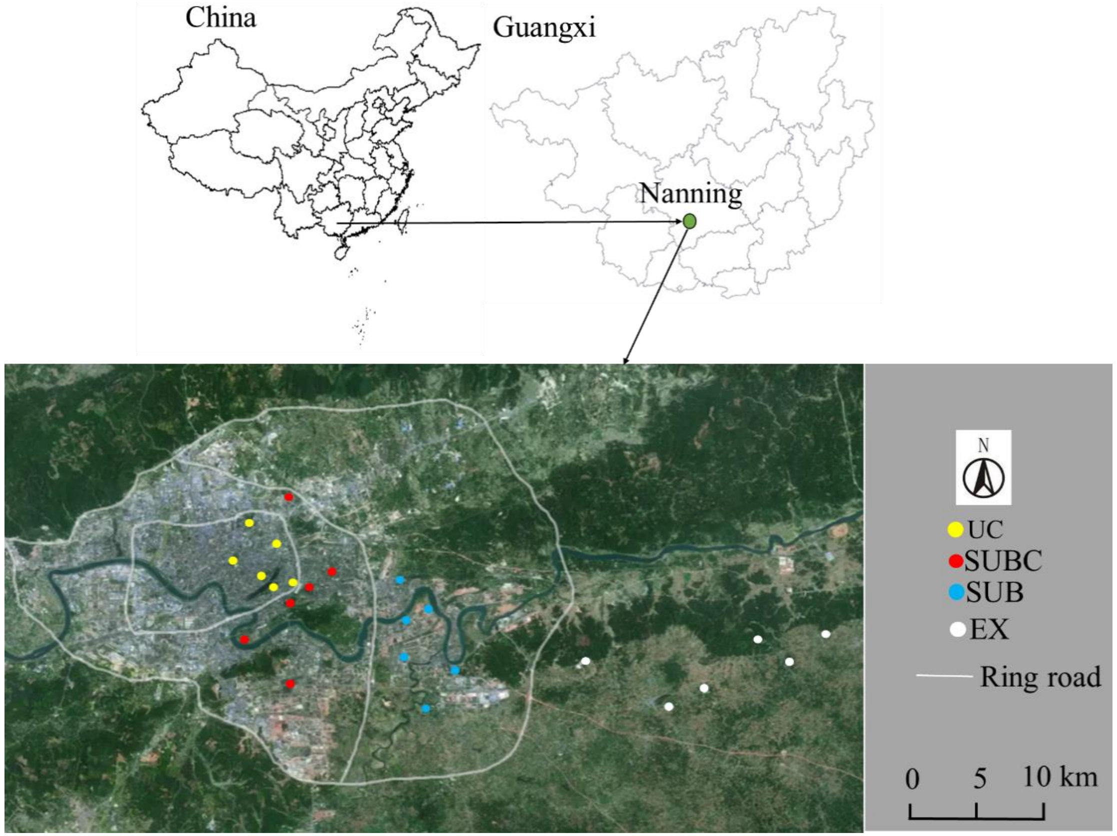

Figure S1. Sampling sites of urban forests in east of Nanning

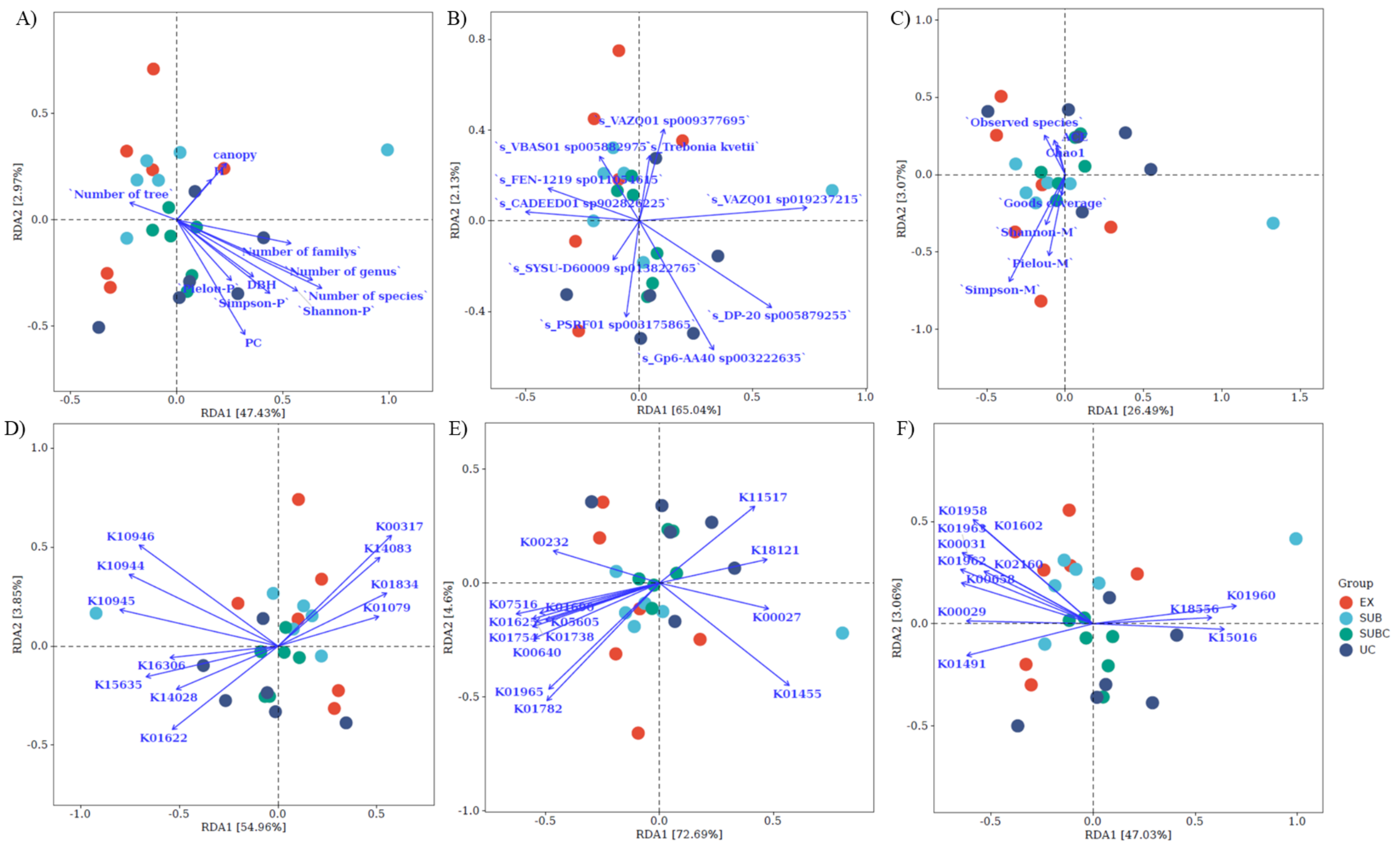

Figure S2 RDA analysis between (A) plant factors, (B) microbial diversity, (C) microbial community, (D) methane metabolism genes, (E) carbon metabolism genes, (F) carbon fixation genes and SOC factors.

Table S1 Tree species structural characteristics of urban forest in eastern of Nanning city

Family	Genus	Species	Height m	DBH cm	Canopy m^2	Number	Proportion %
Myrtaceae	Eucalyptus	*Eucalyptus robusta*	5.77	9.58	2.19	122	25.79%
		Eucalyptus saligna	7.00	19.10	3.00	20	4.23%
	Syzygium	*Syzygium cumini*	5.75	26.90	9.00	15	3.17%
		Syzygium jambos	4.50	13.21	3.13	3	0.63%
Euphorbiaceae	Bischofia	*Bischofia javanica*	5.38	31.67	11.75	17	3.59%
	Vernicia	*Vernicia montana*	12.00	66.85	125.44	1	0.21%
Leguminosae	Delonix	*Delonix regia*	3.00	26.42	36.00	2	0.42%
	Albizia	*Albizia kalkora*	5.50	13.53	4.00	2	0.42%
	Acacia	*Acacia confusa*	7.00	25.78	4.00	2	0.42%
	Cassia	*Cassia siamea*	11.67	17.40	4.50	33	6.98%
	Bauhinia	*Bauhinia purpurea*	4.63	18.96	4.75	37	7.82%
Saxifragaceae	Hydrangea	*Hydrangea vinicolor*	4.00	14.32	3.00	4	0.85%
Apocynaceae	Alstonia	*Alstonia scholaris*	6.00	10.82	1.00	1	0.21%
Meliaceae	Melia	*Melia azedarach*	6.64	16.28	4.71	11	2.33%
Verbenaceae	Tectona	*Tectona grandis*	7.50	22.44	5.00	9	1.90%
Bombacaceae	Ceiba	*Ceiba pentandra*	5.00	30.88	6.00	2	0.42%
Magnoliaceae	Michelia	*Michelia alba*	9.75	31.67	14.50	2	0.42%
Oleaceae	Osmanthus	*Osmanthus fragrans* var. thunbergii	3.00	14.32	3.50	6	1.27%
Anacardiaceae	Mangifera	*Mangifera persiciformis*	11.25	33.90	28.75	17	3.59%
		Mangifera indica	6.57	48.06	24.00	7	1.48%
	Dracontomelon	*Dracontomelon duperreanum*	12.00	79.58	36.00	1	0.21%
Lythraceae	Lagerstroemia	*Lagerstroemia speciosa*	4.50	11.78	3.00	6	1.27%
Rosaceae	Padus	*Padus perulata*	6.67	45.09	6.33	5	1.06%

	Cerasus	*Cerasus campanulata*	3.00	14.01	6.00	1	0.21%
Moraceae	Ficus	*Ficus tinctoria*	9.00	28.33	25.00	2	0.42%
		Ficus elastica	7.50	101.86	200.00	1	0.21%
		Ficus benjamina	7.00	50.29	18.00	4	0.85%
		Ficus cyathistipula	3.00	23.87	6.00	1	0.21%
		Ficus virens	3.50	24.35	5.00	2	0.42%
		Ficus concinna	6.13	24.67	22.50	9	1.90%
		Ficus microcarpa	5.95	36.76	12.40	25	5.29%
	Broussonetia	*Broussonetia papyrifera*	11.00	25.25	25.00	3	0.63%
Taxodiaceae	Taxodium	*Taxodium distichum*	3.75	11.14	2.00	22	4.65%
Ebenaceae	Diospyros	*Diospyros kaki*	6.00	20.37	6.00	1	0.21%
Pinaceae	Pinus	*Pinus massoniana*	4.75	17.51	7.63	4	0.85%
		Pinus echinata	7.50	44.56	13.50	2	0.42%
Sapindaceae	Dimocarpus	*Dimocarpus longan*	7.50	34.70	9.00	4	0.85%
	Litchi	*Litchi chinensis*	5.00	17.19	3.08	6	1.27%
Araliaceae	Heteropanax	*Heteropanax fragrans*	9.00	19.10	4.00	3	0.63%
Ulmaceae	Celtis	*Celtis sinensis*	8.00	20.69	7.50	4	0.85%
Lauraceae	Persea	*Persea americana*	10.00	52.84	25.00	1	0.21%
Palmae	Areca	*Areca triandra*	5.00	23.87	1.00	9	1.90%
	Phoenix	*Phoenix sylvestris*	6.00	33.26	7.63	6	1.27%
	Archontophoenix	*Archontophoenix alexandrae*	6.00	27.59	2.00	10	2.11%
	Livistona	*Livistona chinensis*	5.50	29.68	4.00	5	1.06%
	Roystonea	*Roystonea regia*	11.50	38.04	52.93	15	3.17%
	Elaeis	*Elaeis guineensis*	6.00	25.46	6.00	3	0.63%
	Caryota	*Caryota mitis*	5.75	10.35	1.00	4	0.85%
	Trachycarpus	*Trachycarpus fortunei*	4.00	9.55	2.25	1	0.21%

Table S2 Pearson's correlation between tree species characteristics and microbial diversity index

	Number of trees	Number of family	Number of genus	Number of species	Shannon-P	Pielou-P	Height m	DBH cm	Canopy m^2	PC kg/ m^2
Chao1	-0.069	0.369	0.273	0.275	0.301	0.285	0.161	0.346	0.249	0.271
Goods_coverage	0.113	-0.365	-0.289	-0.300	-0.332	-0.326	-0.163	-0.402	-0.276	-0.283
Simpson-M	0.057	-0.401	-0.339	-0.264	-0.325	-0.323	0.013	-0.212	-0.141	-0.204
Pielou-M	-0.028	0.023	0.049	0.084	0.038	-0.002	0.182	0.063	0.032	0.086
Shannon-M	-0.033	0.134	0.123	0.151	0.122	0.084	0.193	0.153	0.103	0.155
Observed species	-0.039	0.344	0.238	0.237	0.263	0.246	0.141	0.297	0.226	0.244
ACE	-0.069	0.362	0.264	0.267	0.296	0.282	0.150	0.344	0.245	0.266

Table S3 Pearson's correlation between tree species characteristics and microbial community

	Number of trees	Number of family	Number of genus	Number of species	Shannon-P	Pielou-P	Height m	DBH cm	Canopy m^2	PC kg/ m^2
CADEED01 sp902826225	-0.001	-0.074	-0.099	-0.012	0.015	-0.008	-0.232	-0.161	-0.159	-0.195
DP-20 sp005879255	-0.337	**0.440***	**0.463***	**0.460***	**0.464***	**0.409***	0.221	**0.551****	**0.466***	0.318
Gp6-AA40 sp003222635	0.037	-0.083	0.372	0.351	0.337	0.324	0.199	0.355	0.199	**0.621****
VAZQ01 sp009377695	-0.036	0.223	-0.014	0.004	0.060	0.194	-0.009	0.066	-0.111	-0.060
PSRF01 sp003175865	0.260	-0.023	0.169	0.173	0.217	0.338	0.280	0.148	0.145	0.353
SYSU-D60009 sp013822765	-0.298	0.158	0.054	0.146	0.225	0.250	-0.020	0.369	0.134	0.113
FEN-1219 sp011054615	-0.168	0.308	0.159	0.192	0.255	0.218	-0.145	-0.011	-0.036	-0.090
Trebonia kvetii	0.304	-0.339	-0.355	-0.360	-0.389	**-0.417***	-0.271	**-0.472***	-0.230	**-0.429***
VBAS01 sp005882975	0.053	-0.028	-0.098	-0.082	-0.126	-0.283	0.165	0.024	0.017	0.077
VAZQ01 sp019237215	-0.130	0.235	0.284	0.337	0.282	0.218	0.148	-0.007	0.065	-0.053

Table S4 Pearson's correlation between tree species characteristics and methane metabolism genes

	Number of trees	Number of family	Number of genus	Number of species	Shannon-P	Pielou-P	Height m	DBH cm	Canopy m^2	PC kg/ m^2
K00317	-0.006	-0.264	**-0.462***	**-0.465***	**-0.442***	-0.282	-0.064	-0.288	-0.181	**-0.413***
K01079	0.238	**-0.481***	**-0.423***	-0.348	-0.347	-0.329	-0.269	-0.379	-0.233	-0.317
K01622	-0.302	**0.464***	**0.585****	**0.569****	**0.593****	**0.536****	0.128	**0.603****	0.241	**0.538****
K01834	0.088	-0.183	-0.312	-0.272	-0.236	-0.187	-0.081	-0.124	-0.172	-0.081
K10944	0.008	0.140	0.283	0.304	0.212	0.124	0.394	0.267	**0.507***	0.150
K10945	0.009	0.166	0.297	0.325	0.233	0.121	0.305	0.226	0.333	0.134
K10946	-0.038	0.049	0.202	0.224	0.118	0.022	0.373	0.235	**0.467***	0.110
K14028	-0.076	0.215	**0.515****	**0.495***	0.375	0.098	0.209	**0.483***	0.230	0.688**
K14083	0.237	-0.340	-0.364	-0.346	-0.352	-0.236	0.055	-0.171	-0.124	-0.116
K15635	-0.213	**0.434***	**0.597****	**0.584****	**0.531****	**0.453***	0.312	**0.482***	0.395	**0.439***
K16306	-0.213	0.225	0.282	0.275	0.233	0.287	**0.449***	**0.537****	**0.685****	0.324

Table S5 Pearson's correlation between tree species characteristics and carbon metabolism genes

	Number of trees	Number of family	Number of genus	Number of species	Shannon-P	Pielou-P	Height m	DBH cm	Canopy m^2	PC kg/ m^2
K00027	-0.018	0.089	0.282	0.257	0.243	0.259	0.179	0.063	-0.015	0.128
K00232	0.245	-0.186	-0.201	-0.132	-0.095	-0.141	-0.079	**-0.445***	-0.334	-0.395
K00640	0.013	-0.288	-0.354	-0.319	-0.321	-0.299	-0.228	-0.225	-0.165	-0.241
K01455	-0.131	0.081	0.102	0.145	0.120	0.100	0.145	-0.072	-0.135	-0.094
K01625	0.320	**-0.408***	**-0.489***	**-0.461***	-0.384	-0.279	-0.266	**-0.505***	**-0.490***	**-0.421***
K01690	0.060	-0.280	-0.310	-0.221	-0.230	-0.263	-0.172	-0.146	-0.183	-0.115
K01738	0.029	-0.293	-0.297	-0.279	-0.326	-0.307	0.018	-0.233	-0.079	-0.097
K01754	0.048	-0.150	-0.219	-0.149	-0.111	-0.109	-0.030	-0.116	-0.202	-0.156
K01782	-0.050	-0.187	-0.335	-0.325	-0.307	-0.153	-0.016	-0.202	-0.018	-0.294
K01965	0.160	-0.399	**-0.476***	**-0.405***	**-0.416***	**-0.411***	-0.079	-0.330	-0.279	-0.303
K05605	0.328	-0.353	**-0.556****	**-0.533****	**-0.491***	-0.378	-0.217	-0.366	-0.250	-0.370
K07516	0.242	-0.230	-0.266	-0.216	-0.188	-0.167	-0.196	-0.302	-0.269	-0.201
K11517	-0.050	**0.409***	**0.556****	**0.551****	**0.512***	0.388	0.215	**0.456***	0.284	**0.454***
K18121	-0.327	**0.478***	**0.437***	0.396	**0.418***	**0.438***	0.142	**0.541****	**0.489***	0.329

Table S6 Pearson's correlation between tree species characteristics and carbon fixation genes

	Number of trees	Number of family	Number of genus	Number of species	Shannon-P	Pielou-P	Height m	DBH cm	Canopy m^2	PC kg/m^2
K00029	-0.092	-0.127	-0.154	-0.119	-0.129	-0.170	-0.121	-0.125	-0.154	-0.089
K00031	0.103	-0.380	**-0.409***	-0.369	**-0.429***	**-0.477***	-0.030	-0.261	-0.209	-0.179
K00658	0.111	-0.218	-0.257	-0.198	-0.228	-0.272	-0.049	-0.192	-0.168	-0.164
K01491	0.264	-0.096	-0.073	-0.037	-0.076	-0.236	-0.330	-0.309	-0.213	-0.224
K01602	-0.062	-0.372	**-0.454***	**-0.460***	**-0.419***	-0.286	-0.245	-0.362	-0.262	**-0.410***
K01958	-0.022	-0.305	**-0.504***	**-0.511***	**-0.526****	-0.399	-0.062	-0.244	-0.098	-0.278
K01960	-0.271	0.305	**0.533****	**0.519****	**0.456***	0.334	0.207	0.400	0.356	0.334
K01962	0.110	-0.320	-0.377	-0.347	**-0.412***	**-0.486***	-0.216	-0.286	-0.201	0-.245
K01963	0.218	-0.338	-0.319	-0.280	-0.336	**-0.420***	-0.076	-0.361	-0.204	-0.257
K02160	0.134	-0.287	-0.393	-0.370	-0.396	**-0.422***	-0.210	**-0.413***	-0.269	-0.349
K15016	-0.144	0.169	**0.544****	**0.531****	**0.439***	0.335	0.294	**0.465***	**0.427***	**0.501***
K18556	0.164	0.161	**0.427***	**0.448***	0.403	0.378	0.219	0.243	0.194	0.277
